# Supplementary material for: Hot spot prediction in protein-protein interactions by an ensemble system
Source: BMC Syst Biol. 2018 Dec 31;12(Suppl 9):132. doi: 10.1186/s12918-018-0665-8 (PMC6311905; doi:10.1186/s12918-018-0665-8)
Supplement: Supplementary file 2 — All relevant MATLAB code. A simple Matlab implement of our predictor. (ZIP 19,742 kb) [file 12918_2018_665_MOESM2_ESM.zip › Additional file 2/Read ASA/ASA website/dbMPIKT/4IOFA.html]

 NetSurfP - Protein Surface Accessibility and Secondary Structure Predictions

  

|  |  |  |
| --- | --- | --- |
|  |  | NetSurfP - Protein Surface Accessibility and Secondary Structure Predictions  Technical University of Denmark |

  


---

```
# For publication of results, please cite:
# A generic method for assignment of reliability scores applied to solvent accessibility predictions.
# Bent Petersen, Thomas Nordahl Petersen, Pernille Andersen, Morten Nielsen and Claus Lundegaard
# BMC Structural Biology 2009, 9:51 doi:10.1186/1472-6807-9-51
#
# Column 1: Class assignment - B for buried or E for Exposed - Threshold: 25% exposure, but not based on RSA
# Column 2: Amino acid
# Column 3: Sequence name
# Column 4: Amino acid number
# Column 5: Relative Surface Accessibility - RSA
# Column 6: Absolute Surface Accessibility
# Column 7: Z-fit score for RSA prediction
# Column 8: Probability for Alpha-Helix
# Column 9: Probability for Beta-strand
# Column 10: Probability for Coil
E M  4IOFA                  1    0.768 153.637  -1.970   0.003   0.003   0.994
E H  4IOFA                  2    0.546  99.281  -0.866   0.018   0.019   0.964
E H  4IOFA                  3    0.564 102.683  -0.359   0.257   0.016   0.727
E H  4IOFA                  4    0.321  58.463  -0.830   0.694   0.003   0.303
B H  4IOFA                  5    0.269  48.858  -0.659   0.782   0.003   0.216
E H  4IOFA                  6    0.527  95.934  -0.485   0.858   0.002   0.139
B H  4IOFA                  7    0.230  41.855  -0.331   0.879   0.010   0.111
B A  4IOFA                  8    0.131  14.381  -0.610   0.802   0.014   0.185
B A  4IOFA                  9    0.122  13.466  -1.362   0.802   0.014   0.185
E G  4IOFA                 10    0.332  26.136  -0.556   0.717   0.014   0.269
B I  4IOFA                 11    0.212  39.165   0.716   0.522   0.016   0.462
B P  4IOFA                 12    0.244  34.666  -0.567   0.455   0.046   0.498
B M  4IOFA                 13    0.243  48.564  -1.195   0.354   0.048   0.598
E N  4IOFA                 14    0.418  61.151  -1.224   0.268   0.043   0.689
E N  4IOFA                 15    0.367  53.743  -0.202   0.268   0.043   0.689
E P  4IOFA                 16    0.377  53.454  -1.021   0.339   0.016   0.645
B A  4IOFA                 17    0.210  23.164  -0.957   0.430   0.016   0.555
B I  4IOFA                 18    0.134  24.735  -0.557   0.561   0.047   0.393
E K  4IOFA                 19    0.554 114.020   0.693   0.455   0.046   0.498
E R  4IOFA                 20    0.521 119.309   0.200   0.455   0.046   0.498
B I  4IOFA                 21    0.185  34.225   0.091   0.354   0.048   0.598
E G  4IOFA                 22    0.462  36.359  -0.354   0.257   0.016   0.727
E N  4IOFA                 23    0.562  82.350   0.062   0.354   0.048   0.598
E H  4IOFA                 24    0.634 115.416   0.866   0.278   0.093   0.628
B I  4IOFA                 25    0.166  30.691   0.098   0.199   0.152   0.649
E T  4IOFA                 26    0.382  52.983  -0.031   0.191   0.086   0.723
E K  4IOFA                 27    0.533 109.741   0.488   0.113   0.043   0.844
B S  4IOFA                 28    0.233  27.331  -0.898   0.053   0.043   0.903
E P  4IOFA                 29    0.478  67.857  -1.161   0.053   0.043   0.903
E E  4IOFA                 30    0.527  92.119  -1.129   0.053   0.043   0.903
E D  4IOFA                 31    0.507  73.001  -1.181   0.018   0.019   0.964
E K  4IOFA                 32    0.486  99.929  -0.591   0.018   0.019   0.964
E R  4IOFA                 33    0.572 131.102  -0.241   0.018   0.088   0.893
E E  4IOFA                 34    0.509  88.957   1.109   0.005   0.336   0.660
B Y  4IOFA                 35    0.103  22.032   1.038   0.002   0.816   0.182
E R  4IOFA                 36    0.427  97.737   1.028   0.001   0.959   0.040
E G  4IOFA                 37    0.395  31.071   1.023   0.001   0.959   0.040
B L  4IOFA                 38    0.143  26.147   1.240   0.001   0.959   0.040
E E  4IOFA                 39    0.432  75.401   1.166   0.001   0.959   0.040
B L  4IOFA                 40    0.064  11.718   0.477   0.003   0.718   0.279
E A  4IOFA                 41    0.739  81.482   1.014   0.004   0.085   0.910
E N  4IOFA                 42    0.335  49.029  -1.391   0.005   0.015   0.979
E G  4IOFA                 43    0.302  23.791  -0.298   0.004   0.138   0.858
B I  4IOFA                 44    0.043   7.863   0.601   0.004   0.616   0.381
B K  4IOFA                 45    0.259  53.297   1.856   0.001   0.959   0.040
B V  4IOFA                 46    0.032   4.857   1.147   0.000   0.983   0.017
B L  4IOFA                 47    0.047   8.587   1.414   0.000   0.983   0.017
B L  4IOFA                 48    0.037   6.793   0.776   0.000   0.983   0.017
B I  4IOFA                 49    0.111  20.627   1.320   0.000   0.983   0.017
E S  4IOFA                 50    0.320  37.481   0.560   0.001   0.900   0.099
B D  4IOFA                 51    0.152  21.903   0.872   0.004   0.197   0.799
E P  4IOFA                 52    0.658  93.299  -0.710   0.005   0.015   0.979
E T  4IOFA                 53    0.706  97.978   0.076   0.005   0.015   0.979
B T  4IOFA                 54    0.161  22.372   0.022   0.005   0.015   0.979
E D  4IOFA                 55    0.449  64.701   0.081   0.004   0.085   0.910
B K  4IOFA                 56    0.216  44.349   1.051   0.002   0.816   0.182
B S  4IOFA                 57    0.022   2.555   0.729   0.001   0.959   0.040
B S  4IOFA                 58    0.052   6.130  -0.048   0.001   0.959   0.040
B A  4IOFA                 59    0.025   2.777   0.352   0.001   0.959   0.040
B A  4IOFA                 60    0.066   7.251   0.237   0.001   0.959   0.040
B L  4IOFA                 61    0.019   3.534   1.188   0.000   0.983   0.017
B D  4IOFA                 62    0.063   9.021   0.481   0.000   0.983   0.017
B V  4IOFA                 63    0.040   6.209   0.153   0.001   0.959   0.040
B H  4IOFA                 64    0.151  27.376  -0.327   0.003   0.718   0.279
B I  4IOFA                 65    0.062  11.526   0.056   0.004   0.420   0.576
B G  4IOFA                 66    0.067   5.234  -1.442   0.005   0.336   0.660
B S  4IOFA                 67    0.113  13.279  -1.243   0.020   0.205   0.775
B L  4IOFA                 68    0.236  43.212  -0.625   0.018   0.088   0.893
E S  4IOFA                 69    0.416  48.697  -0.260   0.018   0.047   0.935
B D  4IOFA                 70    0.155  22.393  -0.034   0.018   0.019   0.964
E P  4IOFA                 71    0.499  70.879  -0.543   0.058   0.017   0.925
E P  4IOFA                 72    0.552  78.329  -0.142   0.053   0.005   0.942
E N  4IOFA                 73    0.747 109.390   0.464   0.016   0.005   0.979
E I  4IOFA                 74    0.341  63.011   0.449   0.053   0.005   0.942
E A  4IOFA                 75    0.365  40.245   0.626   0.181   0.016   0.803
B G  4IOFA                 76    0.053   4.187  -0.504   0.257   0.016   0.727
B L  4IOFA                 77    0.045   8.276   0.864   0.802   0.014   0.185
B S  4IOFA                 78    0.038   4.465  -0.535   0.923   0.002   0.076
B H  4IOFA                 79    0.096  17.499   0.389   0.970   0.001   0.030
B F  4IOFA                 80    0.036   7.245   0.400   0.970   0.001   0.030
B L  4IOFA                 81    0.044   8.056   0.713   0.970   0.001   0.030
B E  4IOFA                 82    0.041   7.163  -0.616   0.988   0.000   0.012
B H  4IOFA                 83    0.065  11.860   0.236   0.970   0.001   0.030
B M  4IOFA                 84    0.036   7.184   0.392   0.970   0.001   0.030
B L  4IOFA                 85    0.043   7.892   0.511   0.923   0.002   0.076
B F  4IOFA                 86    0.126  25.228  -0.936   0.782   0.003   0.216
B L  4IOFA                 87    0.203  37.114   0.981   0.502   0.002   0.495
B G  4IOFA                 88    0.153  12.033  -1.877   0.176   0.004   0.820
B T  4IOFA                 89    0.082  11.346  -1.288   0.109   0.005   0.886
E K  4IOFA                 90    0.607 124.880   0.865   0.058   0.017   0.925
E K  4IOFA                 91    0.624 128.336   1.122   0.018   0.019   0.964
B Y  4IOFA                 92    0.144  30.837   1.565   0.005   0.015   0.979
E P  4IOFA                 93    0.463  65.686  -0.801   0.005   0.015   0.979
E K  4IOFA                 94    0.535 109.967   0.146   0.016   0.005   0.979
E E  4IOFA                 95    0.479  83.751  -0.637   0.694   0.003   0.303
E N  4IOFA                 96    0.579  84.707  -0.805   0.782   0.003   0.216
E E  4IOFA                 97    0.348  60.813   1.462   0.923   0.002   0.076
B Y  4IOFA                 98    0.030   6.496   0.424   0.970   0.001   0.030
B S  4IOFA                 99    0.211  24.717   1.130   0.970   0.001   0.030
E Q  4IOFA                100    0.528  94.354   1.774   0.970   0.001   0.030
B F  4IOFA                101    0.153  30.667   1.517   0.988   0.000   0.012
B L  4IOFA                102    0.031   5.694   0.224   0.970   0.001   0.030
E S  4IOFA                103    0.523  61.307   0.949   0.970   0.001   0.030
E E  4IOFA                104    0.600 104.803   0.517   0.923   0.002   0.076
B H  4IOFA                105    0.151  27.540   0.348   0.694   0.003   0.303
B A  4IOFA                106    0.215  23.726  -1.317   0.176   0.004   0.820
B G  4IOFA                107    0.118   9.294  -1.698   0.018   0.019   0.964
B S  4IOFA                108    0.261  30.648  -0.734   0.118   0.150   0.732
B S  4IOFA                109    0.094  11.017   0.036   0.069   0.386   0.545
B N  4IOFA                110    0.071  10.438  -0.525   0.069   0.386   0.545
B A  4IOFA                111    0.037   4.110  -1.678   0.069   0.386   0.545
B F  4IOFA                112    0.161  32.393  -1.249   0.069   0.386   0.545
B T  4IOFA                113    0.062   8.586  -0.957   0.069   0.386   0.545
B S  4IOFA                114    0.149  17.428  -0.531   0.056   0.142   0.802
E G  4IOFA                115    0.316  24.869   0.233   0.052   0.084   0.864
B E  4IOFA                116    0.259  45.265  -1.311   0.020   0.205   0.775
B H  4IOFA                117    0.204  37.053   0.619   0.021   0.451   0.528
B T  4IOFA                118    0.027   3.717  -0.572   0.018   0.846   0.136
B N  4IOFA                119    0.095  13.835   0.829   0.011   0.918   0.071
B Y  4IOFA                120    0.052  11.177   0.544   0.006   0.962   0.032
B Y  4IOFA                121    0.132  28.251   0.232   0.006   0.962   0.032
B F  4IOFA                122    0.047   9.453  -0.443   0.001   0.900   0.099
E D  4IOFA                123    0.428  61.603   1.150   0.002   0.816   0.182
B V  4IOFA                124    0.067  10.283   0.390   0.005   0.336   0.660
B S  4IOFA                125    0.198  23.147   0.583   0.018   0.047   0.935
E H  4IOFA                126    0.407  74.106   0.638   0.694   0.003   0.303
E E  4IOFA                127    0.720 125.784   1.561   0.858   0.002   0.139
B H  4IOFA                128    0.206  37.526   0.651   0.694   0.003   0.303
B L  4IOFA                129    0.023   4.138   1.211   0.923   0.002   0.076
E E  4IOFA                130    0.417  72.815   1.238   0.970   0.001   0.030
E G  4IOFA                131    0.407  32.007   1.436   0.970   0.001   0.030
B A  4IOFA                132    0.029   3.141   0.587   0.970   0.001   0.030
B L  4IOFA                133    0.039   7.068   0.517   0.970   0.001   0.030
E D  4IOFA                134    0.355  51.127   1.153   0.970   0.001   0.030
B R  4IOFA                135    0.122  27.984   1.322   0.970   0.001   0.030
B F  4IOFA                136    0.042   8.369   0.789   0.970   0.001   0.030
B A  4IOFA                137    0.059   6.535   0.684   0.970   0.001   0.030
B Q  4IOFA                138    0.266  47.525   0.022   0.970   0.001   0.030
B F  4IOFA                139    0.112  22.438   0.822   0.970   0.001   0.030
B F  4IOFA                140    0.031   6.262  -0.837   0.923   0.002   0.076
E L  4IOFA                141    0.258  47.331   0.062   0.694   0.003   0.303
E S  4IOFA                142    0.399  46.739   0.291   0.246   0.004   0.750
B P  4IOFA                143    0.094  13.268  -0.183   0.109   0.005   0.886
E L  4IOFA                144    0.408  74.650   0.438   0.018   0.019   0.964
B F  4IOFA                145    0.049   9.854   0.528   0.005   0.015   0.979
E D  4IOFA                146    0.425  61.315   2.243   0.003   0.003   0.994
E E  4IOFA                147    0.588 102.724   1.030   0.858   0.002   0.139
E S  4IOFA                148    0.734  86.060   1.091   0.858   0.002   0.139
E A  4IOFA                149    0.265  29.159   1.556   0.858   0.002   0.139
B K  4IOFA                150    0.055  11.334   1.075   0.970   0.001   0.030
E D  4IOFA                151    0.454  65.393   1.629   0.970   0.001   0.030
E R  4IOFA                152    0.497 113.836   2.044   0.970   0.001   0.030
B E  4IOFA                153    0.086  14.972   0.278   0.970   0.001   0.030
B V  4IOFA                154    0.173  26.544   1.419   0.988   0.000   0.012
E N  4IOFA                155    0.524  76.728   1.284   0.970   0.001   0.030
B A  4IOFA                156    0.158  17.434   1.725   0.988   0.000   0.012
B V  4IOFA                157    0.034   5.287   0.410   0.970   0.001   0.030
E D  4IOFA                158    0.294  42.365   1.313   0.988   0.000   0.012
E S  4IOFA                159    0.356  41.723   0.500   0.988   0.000   0.012
B E  4IOFA                160    0.102  17.750  -0.911   0.988   0.000   0.012
B H  4IOFA                161    0.203  36.871   0.865   0.970   0.001   0.030
E E  4IOFA                162    0.483  84.398   0.816   0.923   0.002   0.076
E K  4IOFA                163    0.459  94.499  -0.034   0.782   0.003   0.216
E N  4IOFA                164    0.357  52.323  -0.340   0.502   0.002   0.495
E V  4IOFA                165    0.391  60.035  -0.356   0.246   0.004   0.750
E M  4IOFA                166    0.615 123.001  -1.347   0.058   0.017   0.925
E N  4IOFA                167    0.363  53.187  -0.397   0.003   0.003   0.994
E D  4IOFA                168    0.416  59.946  -0.883   0.694   0.003   0.303
E A  4IOFA                169    0.458  50.472  -0.723   0.923   0.002   0.076
E W  4IOFA                170    0.310  74.651  -0.350   0.923   0.002   0.076
B R  4IOFA                171    0.141  32.381   1.084   0.970   0.001   0.030
B L  4IOFA                172    0.037   6.683  -0.191   0.970   0.001   0.030
B F  4IOFA                173    0.162  32.513   0.644   0.970   0.001   0.030
E Q  4IOFA                174    0.332  59.367   0.475   0.970   0.001   0.030
B L  4IOFA                175    0.152  27.795   1.029   0.970   0.001   0.030
B E  4IOFA                176    0.036   6.237  -0.410   0.970   0.001   0.030
B K  4IOFA                177    0.226  46.509   0.887   0.970   0.001   0.030
E A  4IOFA                178    0.441  48.631   0.619   0.923   0.002   0.076
B T  4IOFA                179    0.137  19.044  -0.507   0.858   0.002   0.139
B G  4IOFA                180    0.054   4.250  -0.422   0.600   0.003   0.397
B N  4IOFA                181    0.177  25.884   0.315   0.109   0.005   0.886
E P  4IOFA                182    0.579  82.188  -0.585   0.016   0.005   0.979
E K  4IOFA                183    0.668 137.366  -1.483   0.005   0.015   0.979
B H  4IOFA                184    0.126  22.901   0.274   0.005   0.015   0.979
B P  4IOFA                185    0.189  26.876  -1.423   0.018   0.047   0.935
B F  4IOFA                186    0.117  23.402  -0.697   0.018   0.047   0.935
B S  4IOFA                187    0.239  27.952  -1.757   0.018   0.047   0.935
E K  4IOFA                188    0.300  61.689  -0.166   0.018   0.047   0.935
B F  4IOFA                189    0.229  45.940  -1.275   0.018   0.019   0.964
B G  4IOFA                190    0.155  12.167  -2.420   0.018   0.047   0.935
B T  4IOFA                191    0.256  35.438  -1.785   0.005   0.045   0.951
B G  4IOFA                192    0.149  11.687  -1.640   0.005   0.045   0.951
E N  4IOFA                193    0.325  47.653   0.556   0.018   0.019   0.964
E K  4IOFA                194    0.369  75.903   0.571   0.782   0.003   0.216
E Y  4IOFA                195    0.668 142.752   0.600   0.782   0.003   0.216
B T  4IOFA                196    0.163  22.664   0.472   0.782   0.003   0.216
B L  4IOFA                197    0.023   4.230   0.658   0.782   0.003   0.216
E E  4IOFA                198    0.508  88.748   1.382   0.694   0.003   0.303
E T  4IOFA                199    0.586  81.278   0.993   0.502   0.002   0.495
B R  4IOFA                200    0.078  17.793   0.147   0.502   0.002   0.495
E P  4IOFA                201    0.425  60.237  -0.503   0.502   0.002   0.495
E N  4IOFA                202    0.563  82.379  -0.621   0.502   0.002   0.495
E Q  4IOFA                203    0.514  91.800  -1.223   0.406   0.004   0.590
E E  4IOFA                204    0.470  82.039  -1.296   0.246   0.004   0.750
E G  4IOFA                205    0.514  40.491  -0.988   0.176   0.004   0.820
E I  4IOFA                206    0.426  78.847  -0.630   0.406   0.004   0.590
E D  4IOFA                207    0.432  62.280   0.224   0.522   0.016   0.462
B V  4IOFA                208    0.083  12.680   0.199   0.717   0.014   0.269
B R  4IOFA                209    0.159  36.434   0.957   0.970   0.001   0.030
E Q  4IOFA                210    0.599 106.910   0.926   0.970   0.001   0.030
E E  4IOFA                211    0.248  43.238   1.704   0.970   0.001   0.030
B L  4IOFA                212    0.011   2.069   1.751   0.988   0.000   0.012
E L  4IOFA                213    0.287  52.568   1.793   0.988   0.000   0.012
E K  4IOFA                214    0.545 112.045   2.058   0.988   0.000   0.012
B F  4IOFA                215    0.093  18.585   1.224   0.970   0.001   0.030
B H  4IOFA                216    0.096  17.426   1.052   0.970   0.001   0.030
E S  4IOFA                217    0.550  64.507   0.872   0.923   0.002   0.076
E A  4IOFA                218    0.571  62.869   1.560   0.858   0.002   0.139
B Y  4IOFA                219    0.241  51.502   0.560   0.622   0.015   0.363
B Y  4IOFA                220    0.083  17.694   0.122   0.257   0.016   0.727
B S  4IOFA                221    0.293  34.375   0.725   0.058   0.017   0.925
B S  4IOFA                222    0.113  13.244  -0.566   0.058   0.017   0.925
E N  4IOFA                223    0.508  74.400  -0.106   0.018   0.019   0.964
B L  4IOFA                224    0.147  26.952   0.624   0.004   0.138   0.858
B M  4IOFA                225    0.019   3.822   1.057   0.003   0.718   0.279
B A  4IOFA                226    0.051   5.598   1.364   0.001   0.959   0.040
B V  4IOFA                227    0.016   2.413   1.554   0.000   0.983   0.017
B V  4IOFA                228    0.030   4.534   0.941   0.000   0.983   0.017
B V  4IOFA                229    0.018   2.782   1.029   0.000   0.983   0.017
B L  4IOFA                230    0.056  10.180   0.751   0.001   0.959   0.040
B G  4IOFA                231    0.054   4.266  -0.366   0.007   0.526   0.467
E R  4IOFA                232    0.501 114.843   0.325   0.004   0.085   0.910
B E  4IOFA                233    0.157  27.410   0.605   0.005   0.015   0.979
E S  4IOFA                234    0.469  55.025   1.522   0.003   0.003   0.994
B L  4IOFA                235    0.159  29.058   0.786   0.923   0.002   0.076
E D  4IOFA                236    0.704 101.461   1.392   0.970   0.001   0.030
E D  4IOFA                237    0.406  58.505   2.157   0.970   0.001   0.030
B L  4IOFA                238    0.026   4.816   0.793   0.970   0.001   0.030
B T  4IOFA                239    0.197  27.352   1.829   0.970   0.001   0.030
E N  4IOFA                240    0.581  85.117   2.183   0.970   0.001   0.030
B L  4IOFA                241    0.193  35.320   2.186   0.970   0.001   0.030
B V  4IOFA                242    0.014   2.183   1.581   0.988   0.000   0.012
E V  4IOFA                243    0.433  66.629   2.212   0.970   0.001   0.030
E K  4IOFA                244    0.649 133.561   2.028   0.970   0.001   0.030
B L  4IOFA                245    0.209  38.195   1.248   0.923   0.002   0.076
B F  4IOFA                246    0.057  11.440   0.009   0.694   0.003   0.303
E S  4IOFA                247    0.569  66.722   0.831   0.406   0.004   0.590
E E  4IOFA                248    0.708 123.757   1.479   0.176   0.004   0.820
B V  4IOFA                249    0.097  14.832   1.089   0.053   0.005   0.942
E E  4IOFA                250    0.639 111.686   0.832   0.115   0.016   0.868
E N  4IOFA                251    0.647  94.750   0.206   0.053   0.005   0.942
E K  4IOFA                252    0.553 113.732  -0.472   0.053   0.005   0.942
E N  4IOFA                253    0.763 111.747  -0.980   0.016   0.005   0.979
E V  4IOFA                254    0.424  65.153  -0.642   0.018   0.019   0.964
E P  4IOFA                255    0.475  67.459  -1.036   0.018   0.019   0.964
E L  4IOFA                256    0.427  78.147  -0.256   0.058   0.017   0.925
E P  4IOFA                257    0.507  71.915  -0.616   0.053   0.043   0.903
E E  4IOFA                258    0.538  94.041  -0.557   0.113   0.043   0.844
E F  4IOFA                259    0.333  66.793  -0.344   0.113   0.043   0.844
E P  4IOFA                260    0.426  60.421  -0.956   0.113   0.043   0.844
E E  4IOFA                261    0.610 106.584  -0.847   0.113   0.043   0.844
E H  4IOFA                262    0.486  88.403  -0.511   0.053   0.043   0.903
E P  4IOFA                263    0.336  47.735  -1.260   0.053   0.043   0.903
B F  4IOFA                264    0.245  49.151  -0.934   0.053   0.043   0.903
E Q  4IOFA                265    0.487  87.014  -0.628   0.053   0.043   0.903
E E  4IOFA                266    0.522  91.263  -1.108   0.113   0.043   0.844
E E  4IOFA                267    0.609 106.340  -1.627   0.053   0.043   0.903
E H  4IOFA                268    0.573 104.174  -1.107   0.019   0.141   0.840
E L  4IOFA                269    0.412  75.456  -0.438   0.005   0.336   0.660
E K  4IOFA                270    0.293  60.311   0.007   0.003   0.718   0.279
E Q  4IOFA                271    0.357  63.707   0.436   0.001   0.900   0.099
B L  4IOFA                272    0.237  43.413  -0.126   0.001   0.959   0.040
B Y  4IOFA                273    0.205  43.744   0.063   0.001   0.959   0.040
E K  4IOFA                274    0.327  67.284   0.142   0.001   0.959   0.040
E I  4IOFA                275    0.314  58.090  -0.319   0.001   0.900   0.099
E V  4IOFA                276    0.368  56.623   0.420   0.004   0.616   0.381
E P  4IOFA                277    0.574  81.380  -0.784   0.005   0.262   0.733
E I  4IOFA                278    0.570 105.468  -0.811   0.018   0.088   0.893
E K  4IOFA                279    0.584 120.067  -0.567   0.053   0.043   0.903
E D  4IOFA                280    0.683  98.449   0.557   0.052   0.084   0.864
E I  4IOFA                281    0.231  42.772   0.614   0.066   0.296   0.638
E R  4IOFA                282    0.366  83.906   0.890   0.022   0.552   0.426
B N  4IOFA                283    0.160  23.468   1.753   0.011   0.918   0.071
B L  4IOFA                284    0.019   3.479   1.287   0.001   0.959   0.040
B Y  4IOFA                285    0.062  13.292   1.029   0.000   0.983   0.017
B V  4IOFA                286    0.019   2.997   1.056   0.000   0.983   0.017
B T  4IOFA                287    0.019   2.635   0.335   0.000   0.983   0.017
B F  4IOFA                288    0.045   9.072   0.209   0.001   0.959   0.040
B P  4IOFA                289    0.128  18.121  -0.800   0.003   0.718   0.279
B I  4IOFA                290    0.071  13.098  -0.269   0.004   0.197   0.799
E P  4IOFA                291    0.331  47.040  -0.520   0.005   0.045   0.951
E D  4IOFA                292    0.445  64.067  -1.856   0.018   0.019   0.964
B L  4IOFA                293    0.249  45.629  -0.762   0.058   0.017   0.925
E Q  4IOFA                294    0.499  89.157  -0.844   0.053   0.005   0.942
E K  4IOFA                295    0.605 124.469  -0.718   0.176   0.004   0.820
E Y  4IOFA                296    0.382  81.591  -0.433   0.321   0.003   0.675
E Y  4IOFA                297    0.299  63.960  -0.529   0.782   0.003   0.216
E K  4IOFA                298    0.375  77.137   0.006   0.782   0.003   0.216
E S  4IOFA                299    0.304  35.594  -0.023   0.879   0.010   0.111
B N  4IOFA                300    0.146  21.345   1.003   0.938   0.007   0.055
B P  4IOFA                301    0.035   5.009  -0.742   0.970   0.001   0.030
B G  4IOFA                302    0.076   5.973  -0.525   0.970   0.001   0.030
E H  4IOFA                303    0.300  54.625   1.543   0.970   0.001   0.030
B Y  4IOFA                304    0.041   8.826   0.252   0.988   0.000   0.012
B L  4IOFA                305    0.027   5.035   0.738   0.988   0.000   0.012
B G  4IOFA                306    0.122   9.601  -0.080   0.988   0.000   0.012
B H  4IOFA                307    0.231  42.092   1.019   0.970   0.001   0.030
B L  4IOFA                308    0.045   8.240   0.686   0.970   0.001   0.030
B I  4IOFA                309    0.055  10.267   0.492   0.858   0.002   0.139
E G  4IOFA                310    0.264  20.761  -1.152   0.694   0.003   0.303
E H  4IOFA                311    0.393  71.414  -1.995   0.246   0.004   0.750
B E  4IOFA                312    0.302  52.690  -2.133   0.109   0.005   0.886
E G  4IOFA                313    0.343  27.010  -1.930   0.176   0.004   0.820
B P  4IOFA                314    0.272  38.554  -1.912   0.181   0.016   0.803
B G  4IOFA                315    0.148  11.663  -2.424   0.339   0.016   0.645
E S  4IOFA                316    0.272  31.855   0.152   0.802   0.014   0.185
B L  4IOFA                317    0.046   8.496  -1.177   0.923   0.002   0.076
B L  4IOFA                318    0.125  22.851  -0.083   0.970   0.001   0.030
E S  4IOFA                319    0.432  50.630   0.355   0.970   0.001   0.030
E E  4IOFA                320    0.274  47.780   0.483   0.975   0.003   0.022
B L  4IOFA                321    0.096  17.633   0.231   0.938   0.007   0.055
E K  4IOFA                322    0.279  57.308   0.873   0.938   0.007   0.055
E S  4IOFA                323    0.637  74.656   0.363   0.879   0.010   0.111
E K  4IOFA                324    0.604 124.325   0.451   0.522   0.016   0.462
E G  4IOFA                325    0.589  46.323  -0.908   0.115   0.016   0.868
E W  4IOFA                326    0.310  74.651   0.222   0.053   0.043   0.903
B V  4IOFA                327    0.039   6.071  -0.841   0.064   0.216   0.721
B N  4IOFA                328    0.155  22.677  -0.528   0.022   0.552   0.426
B T  4IOFA                329    0.138  19.113  -1.070   0.023   0.655   0.322
B L  4IOFA                330    0.059  10.821  -0.811   0.022   0.552   0.426
B V  4IOFA                331    0.041   6.348  -0.967   0.023   0.655   0.322
B G  4IOFA                332    0.060   4.753  -2.605   0.022   0.552   0.426
B G  4IOFA                333    0.042   3.274  -2.358   0.021   0.451   0.528
B Q  4IOFA                334    0.102  18.306  -2.084   0.021   0.451   0.528
B K  4IOFA                335    0.123  25.363  -2.259   0.066   0.296   0.638
B E  4IOFA                336    0.201  35.062  -2.042   0.056   0.142   0.802
B G  4IOFA                337    0.204  16.086  -2.383   0.053   0.043   0.903
B A  4IOFA                338    0.207  22.767  -2.210   0.053   0.043   0.903
E R  4IOFA                339    0.393  90.089  -1.403   0.058   0.017   0.925
E G  4IOFA                340    0.350  27.569  -1.674   0.053   0.043   0.903
B F  4IOFA                341    0.127  25.449  -0.760   0.018   0.088   0.893
B M  4IOFA                342    0.065  13.047  -2.053   0.005   0.336   0.660
B F  4IOFA                343    0.065  13.086   0.107   0.002   0.816   0.182
B F  4IOFA                344    0.026   5.278   0.142   0.001   0.900   0.099
B I  4IOFA                345    0.036   6.605  -0.449   0.001   0.959   0.040
B I  4IOFA                346    0.018   3.386   0.829   0.000   0.983   0.017
B N  4IOFA                347    0.046   6.734  -0.571   0.000   0.983   0.017
B V  4IOFA                348    0.024   3.673   0.003   0.001   0.959   0.040
B D  4IOFA                349    0.122  17.609  -0.487   0.001   0.959   0.040
B L  4IOFA                350    0.029   5.347  -0.092   0.002   0.816   0.182
E T  4IOFA                351    0.239  33.122   0.862   0.005   0.336   0.660
E E  4IOFA                352    0.525  91.700  -0.739   0.018   0.088   0.893
E E  4IOFA                353    0.638 111.441  -0.411   0.109   0.005   0.886
E G  4IOFA                354    0.483  38.012  -1.061   0.109   0.005   0.886
E L  4IOFA                355    0.326  59.782  -0.614   0.321   0.003   0.675
E L  4IOFA                356    0.515  94.296   0.265   0.321   0.003   0.675
E H  4IOFA                357    0.337  61.318   1.512   0.181   0.016   0.803
B V  4IOFA                358    0.050   7.716   0.687   0.970   0.001   0.030
E E  4IOFA                359    0.523  91.368   1.349   0.970   0.001   0.030
E D  4IOFA                360    0.464  66.848   2.383   0.988   0.000   0.012
B I  4IOFA                361    0.014   2.535   1.630   0.988   0.000   0.012
B I  4IOFA                362    0.089  16.484   1.328   0.988   0.000   0.012
E L  4IOFA                363    0.520  95.285   2.020   0.970   0.001   0.030
B H  4IOFA                364    0.174  31.632   1.887   0.970   0.001   0.030
B M  4IOFA                365    0.014   2.741   1.725   0.988   0.000   0.012
B F  4IOFA                366    0.225  45.137   1.501   0.970   0.001   0.030
E Q  4IOFA                367    0.499  89.175   1.912   0.970   0.001   0.030
B Y  4IOFA                368    0.088  18.870   1.010   0.970   0.001   0.030
B I  4IOFA                369    0.031   5.809   0.582   0.988   0.000   0.012
E Q  4IOFA                370    0.452  80.691   2.083   0.988   0.000   0.012
E K  4IOFA                371    0.365  75.060   2.214   0.970   0.001   0.030
B L  4IOFA                372    0.022   4.101   0.910   0.970   0.001   0.030
E R  4IOFA                373    0.319  73.005   1.205   0.858   0.002   0.139
E A  4IOFA                374    0.803  88.546   1.469   0.600   0.003   0.397
E E  4IOFA                375    0.435  75.925   1.481   0.109   0.005   0.886
E G  4IOFA                376    0.556  43.765   0.284   0.016   0.005   0.979
B P  4IOFA                377    0.057   8.060   0.125   0.003   0.003   0.994
E Q  4IOFA                378    0.418  74.708   1.902   0.016   0.005   0.979
E E  4IOFA                379    0.555  96.906   1.606   0.923   0.002   0.076
E W  4IOFA                380    0.596 143.242   1.563   0.970   0.001   0.030
B V  4IOFA                381    0.141  21.656   1.428   0.970   0.001   0.030
B F  4IOFA                382    0.048   9.694   1.163   0.970   0.001   0.030
E Q  4IOFA                383    0.483  86.246   1.911   0.970   0.001   0.030
E E  4IOFA                384    0.423  73.846   1.911   0.970   0.001   0.030
B L  4IOFA                385    0.034   6.170   0.281   0.970   0.001   0.030
B K  4IOFA                386    0.177  36.409   1.037   0.970   0.001   0.030
E D  4IOFA                387    0.487  70.220   0.843   0.923   0.002   0.076
B L  4IOFA                388    0.226  41.326   0.983   0.970   0.001   0.030
B N  4IOFA                389    0.092  13.483   0.273   0.970   0.001   0.030
E A  4IOFA                390    0.342  37.699   0.520   0.970   0.001   0.030
B V  4IOFA                391    0.177  27.220  -0.567   0.923   0.002   0.076
B A  4IOFA                392    0.262  28.916  -1.161   0.858   0.002   0.139
B F  4IOFA                393    0.247  49.513  -0.360   0.858   0.002   0.139
E R  4IOFA                394    0.316  72.364   0.729   0.879   0.010   0.111
B F  4IOFA                395    0.235  47.205  -0.359   0.802   0.014   0.185
E K  4IOFA                396    0.384  78.927  -0.454   0.522   0.016   0.462
E D  4IOFA                397    0.484  69.687  -1.236   0.339   0.016   0.645
E K  4IOFA                398    0.471  96.926  -1.087   0.257   0.016   0.727
E E  4IOFA                399    0.479  83.681  -2.023   0.181   0.016   0.803
E R  4IOFA                400    0.444 101.722  -0.619   0.115   0.016   0.868
B P  4IOFA                401    0.213  30.281  -2.422   0.858   0.002   0.139
E R  4IOFA                402    0.361  82.600  -1.384   0.923   0.002   0.076
E G  4IOFA                403    0.424  33.400  -0.498   0.970   0.001   0.030
B Y  4IOFA                404    0.133  28.379   0.601   0.970   0.001   0.030
B T  4IOFA                405    0.029   4.050  -0.778   0.970   0.001   0.030
B S  4IOFA                406    0.221  25.889  -0.652   0.970   0.001   0.030
B K  4IOFA                407    0.240  49.409  -0.066   0.970   0.001   0.030
B I  4IOFA                408    0.038   6.956  -0.381   0.970   0.001   0.030
B A  4IOFA                409    0.037   4.055  -0.682   0.970   0.001   0.030
E G  4IOFA                410    0.303  23.854  -0.097   0.970   0.001   0.030
B I  4IOFA                411    0.118  21.885  -0.538   0.923   0.002   0.076
B L  4IOFA                412    0.076  13.934  -0.603   0.858   0.002   0.139
E H  4IOFA                413    0.308  56.062  -0.950   0.694   0.003   0.303
E Y  4IOFA                414    0.418  89.412  -0.614   0.406   0.004   0.590
B Y  4IOFA                415    0.165  35.239  -0.292   0.058   0.017   0.925
E P  4IOFA                416    0.422  59.868  -0.343   0.016   0.005   0.979
B L  4IOFA                417    0.193  35.412  -0.200   0.321   0.003   0.675
E E  4IOFA                418    0.586 102.357  -0.037   0.600   0.003   0.397
E E  4IOFA                419    0.304  53.039   0.272   0.622   0.015   0.363
B V  4IOFA                420    0.059   9.007  -0.034   0.717   0.014   0.269
B L  4IOFA                421    0.142  26.055  -0.584   0.622   0.015   0.363
E T  4IOFA                422    0.416  57.727   0.673   0.430   0.016   0.555
B A  4IOFA                423    0.090   9.940   0.239   0.858   0.002   0.139
E E  4IOFA                424    0.286  49.877   0.223   0.923   0.002   0.076
E Y  4IOFA                425    0.527 112.727   0.494   0.970   0.001   0.030
B L  4IOFA                426    0.178  32.519   0.988   0.923   0.002   0.076
B L  4IOFA                427    0.037   6.756   0.109   0.858   0.002   0.139
E E  4IOFA                428    0.572  99.893   1.280   0.782   0.003   0.216
E E  4IOFA                429    0.628 109.642   1.884   0.600   0.003   0.397
B F  4IOFA                430    0.060  12.022   0.928   0.246   0.004   0.750
E R  4IOFA                431    0.375  85.967   1.741   0.058   0.017   0.925
E P  4IOFA                432    0.369  52.361   1.492   0.970   0.001   0.030
E D  4IOFA                433    0.716 103.132   1.768   0.970   0.001   0.030
B L  4IOFA                434    0.189  34.588   1.916   0.970   0.001   0.030
B I  4IOFA                435    0.025   4.588   1.184   0.970   0.001   0.030
E E  4IOFA                436    0.338  59.084   2.473   0.970   0.001   0.030
E M  4IOFA                437    0.479  95.748   2.377   0.970   0.001   0.030
B V  4IOFA                438    0.035   5.395   0.691   0.970   0.001   0.030
B L  4IOFA                439    0.043   7.873   0.465   0.970   0.001   0.030
E D  4IOFA                440    0.586  84.385   1.776   0.923   0.002   0.076
E K  4IOFA                441    0.327  67.284   1.842   0.858   0.002   0.139
B L  4IOFA                442    0.033   5.987   0.637   0.694   0.003   0.303
E R  4IOFA                443    0.280  64.051   1.117   0.406   0.004   0.590
E P  4IOFA                444    0.510  72.412  -0.564   0.406   0.004   0.590
E E  4IOFA                445    0.593 103.545  -1.312   0.115   0.016   0.868
E N  4IOFA                446    0.402  58.838   0.010   0.005   0.045   0.951
B V  4IOFA                447    0.077  11.850  -0.079   0.004   0.420   0.576
B R  4IOFA                448    0.128  29.358   0.393   0.001   0.900   0.099
B V  4IOFA                449    0.038   5.764  -0.032   0.001   0.959   0.040
B A  4IOFA                450    0.024   2.634   0.609   0.001   0.959   0.040
B I  4IOFA                451    0.037   6.789  -0.269   0.001   0.959   0.040
B V  4IOFA                452    0.050   7.716  -0.176   0.002   0.816   0.182
B S  4IOFA                453    0.068   7.993  -0.070   0.005   0.336   0.660
E K  4IOFA                454    0.268  55.066   0.004   0.018   0.088   0.893
E S  4IOFA                455    0.550  64.472  -0.526   0.053   0.043   0.903
E F  4IOFA                456    0.454  91.078  -1.388   0.113   0.043   0.844
E E  4IOFA                457    0.405  70.719  -1.304   0.184   0.043   0.773
E G  4IOFA                458    0.428  33.644  -1.505   0.257   0.016   0.727
E K  4IOFA                459    0.588 120.952  -1.502   0.339   0.016   0.645
E T  4IOFA                460    0.429  59.502  -0.459   0.455   0.046   0.498
E D  4IOFA                461    0.406  58.548  -0.459   0.455   0.046   0.498
E R  4IOFA                462    0.472 108.042  -0.373   0.561   0.047   0.393
E T  4IOFA                463    0.426  59.100  -0.170   0.561   0.047   0.393
E E  4IOFA                464    0.439  76.658  -0.964   0.561   0.047   0.393
E E  4IOFA                465    0.367  64.115  -0.314   0.605   0.105   0.290
E W  4IOFA                466    0.361  86.845  -0.900   0.502   0.102   0.396
E Y  4IOFA                467    0.342  73.064  -0.658   0.386   0.097   0.517
E G  4IOFA                468    0.418  32.873  -0.818   0.278   0.093   0.628
B T  4IOFA                469    0.240  33.233  -0.191   0.278   0.093   0.628
E Q  4IOFA                470    0.517  92.354  -0.067   0.386   0.097   0.517
E Y  4IOFA                471    0.312  66.781  -0.198   0.386   0.097   0.517
E K  4IOFA                472    0.411  84.502  -0.470   0.386   0.097   0.517
E Q  4IOFA                473    0.324  57.849  -0.081   0.278   0.093   0.628
E E  4IOFA                474    0.507  88.573   0.057   0.268   0.043   0.689
E A  4IOFA                475    0.616  67.883   0.032   0.268   0.043   0.689
B I  4IOFA                476    0.195  36.057  -0.563   0.339   0.016   0.645
E P  4IOFA                477    0.384  54.447  -0.304   0.522   0.016   0.462
E D  4IOFA                478    0.598  86.129  -0.373   0.782   0.003   0.216
E E  4IOFA                479    0.420  73.304  -0.091   0.782   0.003   0.216
B V  4IOFA                480    0.138  21.149  -0.228   0.858   0.002   0.139
E I  4IOFA                481    0.277  51.171   0.183   0.858   0.002   0.139
E K  4IOFA                482    0.498 102.377   0.092   0.879   0.010   0.111
E K  4IOFA                483    0.347  71.440   0.631   0.879   0.010   0.111
B W  4IOFA                484    0.168  40.452  -0.301   0.802   0.014   0.185
E Q  4IOFA                485    0.439  78.423  -0.209   0.717   0.014   0.269
E N  4IOFA                486    0.460  67.329  -0.311   0.522   0.016   0.462
E A  4IOFA                487    0.388  42.791  -0.858   0.430   0.016   0.555
E D  4IOFA                488    0.535  77.166  -1.145   0.430   0.016   0.555
E L  4IOFA                489    0.364  66.593  -0.408   0.430   0.016   0.555
E N  4IOFA                490    0.364  53.260  -0.753   0.522   0.016   0.462
E G  4IOFA                491    0.538  42.333  -1.291   0.522   0.016   0.462
E K  4IOFA                492    0.499 102.706  -0.955   0.522   0.016   0.462
B F  4IOFA                493    0.192  38.434  -0.738   0.455   0.046   0.498
E K  4IOFA                494    0.438  90.035  -1.224   0.354   0.048   0.598
E L  4IOFA                495    0.389  71.263  -0.830   0.268   0.043   0.689
E P  4IOFA                496    0.323  45.791  -1.212   0.354   0.048   0.598
E T  4IOFA                497    0.458  63.566  -1.436   0.455   0.046   0.498
E K  4IOFA                498    0.460  94.684  -1.406   0.339   0.016   0.645
E N  4IOFA                499    0.387  56.613  -1.576   0.181   0.016   0.803
E E  4IOFA                500    0.554  96.749  -1.066   0.113   0.043   0.844
B F  4IOFA                501    0.301  60.431  -1.744   0.113   0.087   0.800
B I  4IOFA                502    0.257  47.471  -0.660   0.053   0.043   0.903
E P  4IOFA                503    0.367  52.049  -1.408   0.053   0.043   0.903
E T  4IOFA                504    0.506  70.182  -0.825   0.113   0.087   0.800
E N  4IOFA                505    0.630  92.217  -1.643   0.053   0.043   0.903
E F  4IOFA                506    0.350  70.325   0.024   0.053   0.043   0.903
E E  4IOFA                507    0.573 100.173   0.196   0.056   0.142   0.802
B I  4IOFA                508    0.160  29.563   0.315   0.064   0.216   0.721
E L  4IOFA                509    0.442  81.022   0.415   0.066   0.296   0.638
E P  4IOFA                510    0.508  72.142  -0.460   0.064   0.216   0.721
E L  4IOFA                511    0.401  73.478  -0.522   0.056   0.142   0.802
E E  4IOFA                512    0.634 110.812  -1.376   0.052   0.084   0.864
E K  4IOFA                513    0.643 132.306  -1.111   0.053   0.043   0.903
E E  4IOFA                514    0.627 109.589  -1.853   0.053   0.043   0.903
E A  4IOFA                515    0.478  52.632  -1.088   0.053   0.043   0.903
E T  4IOFA                516    0.417  57.782  -0.455   0.056   0.142   0.802
E P  4IOFA                517    0.533  75.576   0.082   0.021   0.279   0.699
B Y  4IOFA                518    0.251  53.532   0.599   0.004   0.420   0.576
B P  4IOFA                519    0.190  26.890  -0.399   0.003   0.718   0.279
B A  4IOFA                520    0.248  27.385   0.562   0.001   0.959   0.040
E L  4IOFA                521    0.322  59.031   0.920   0.001   0.959   0.040
B I  4IOFA                522    0.139  25.660   1.094   0.000   0.983   0.017
E K  4IOFA                523    0.409  84.152   0.288   0.001   0.959   0.040
E D  4IOFA                524    0.338  48.634  -1.046   0.002   0.816   0.182
E T  4IOFA                525    0.494  68.559  -1.307   0.004   0.085   0.910
E A  4IOFA                526    0.604  66.539  -1.581   0.005   0.015   0.979
E M  4IOFA                527    0.329  65.853  -0.846   0.004   0.085   0.910
B S  4IOFA                528    0.063   7.407  -0.288   0.003   0.718   0.279
E K  4IOFA                529    0.265  54.613   0.937   0.001   0.959   0.040
B L  4IOFA                530    0.028   5.108   0.746   0.001   0.959   0.040
B W  4IOFA                531    0.075  17.965   0.934   0.001   0.959   0.040
B F  4IOFA                532    0.083  16.738   0.535   0.001   0.959   0.040
B K  4IOFA                533    0.171  35.195   0.886   0.001   0.959   0.040
E Q  4IOFA                534    0.273  48.669  -0.225   0.003   0.718   0.279
B D  4IOFA                535    0.283  40.766  -0.257   0.004   0.197   0.799
E D  4IOFA                536    0.523  75.321  -1.610   0.004   0.085   0.910
E K  4IOFA                537    0.502 103.261  -0.540   0.005   0.262   0.733
B F  4IOFA                538    0.204  41.003  -0.958   0.004   0.197   0.799
E F  4IOFA                539    0.519 104.103  -0.544   0.004   0.138   0.858
B L  4IOFA                540    0.197  36.052   0.333   0.004   0.420   0.576
B P  4IOFA                541    0.159  22.576  -1.186   0.004   0.616   0.381
B K  4IOFA                542    0.157  32.295   0.564   0.001   0.900   0.099
B A  4IOFA                543    0.031   3.471  -0.812   0.001   0.959   0.040
B N  4IOFA                544    0.085  12.488   0.158   0.000   0.983   0.017
B L  4IOFA                545    0.023   4.175   0.652   0.000   0.983   0.017
B N  4IOFA                546    0.068   9.897   0.154   0.001   0.959   0.040
B F  4IOFA                547    0.021   4.275   0.922   0.001   0.959   0.040
B E  4IOFA                548    0.092  16.125  -0.322   0.001   0.959   0.040
B F  4IOFA                549    0.037   7.326  -0.705   0.001   0.900   0.099
E F  4IOFA                550    0.248  49.774  -0.182   0.004   0.616   0.381
B S  4IOFA                551    0.095  11.099  -1.069   0.004   0.197   0.799
E P  4IOFA                552    0.328  46.557  -1.680   0.005   0.045   0.951
E F  4IOFA                553    0.374  75.102  -1.282   0.018   0.047   0.935
E A  4IOFA                554    0.325  35.837  -1.359   0.053   0.043   0.903
E Y  4IOFA                555    0.495 105.824  -1.575   0.058   0.017   0.925
B V  4IOFA                556    0.250  38.379  -1.271   0.115   0.016   0.868
E D  4IOFA                557    0.397  57.193  -0.941   0.246   0.004   0.750
B P  4IOFA                558    0.280  39.732  -1.409   0.600   0.003   0.397
E L  4IOFA                559    0.285  52.147  -0.754   0.694   0.003   0.303
B H  4IOFA                560    0.173  31.505  -1.312   0.782   0.003   0.216
B S  4IOFA                561    0.158  18.471  -0.250   0.858   0.002   0.139
B N  4IOFA                562    0.058   8.491  -1.358   0.923   0.002   0.076
B M  4IOFA                563    0.059  11.726   0.146   0.970   0.001   0.030
B A  4IOFA                564    0.046   5.113  -0.518   0.970   0.001   0.030
B Y  4IOFA                565    0.212  45.390   0.385   0.988   0.000   0.012
B L  4IOFA                566    0.060  10.968  -0.406   0.970   0.001   0.030
B Y  4IOFA                567    0.063  13.463   0.048   0.988   0.000   0.012
B L  4IOFA                568    0.131  24.059  -0.405   0.988   0.000   0.012
B E  4IOFA                569    0.247  43.081  -0.166   0.970   0.001   0.030
B L  4IOFA                570    0.059  10.803   0.086   0.970   0.001   0.030
B L  4IOFA                571    0.092  16.827  -0.162   0.923   0.002   0.076
E K  4IOFA                572    0.424  87.279  -0.076   0.858   0.002   0.139
E D  4IOFA                573    0.463  66.733   0.172   0.694   0.003   0.303
E S  4IOFA                574    0.313  36.730  -1.135   0.502   0.002   0.495
B L  4IOFA                575    0.135  24.719  -0.256   0.600   0.003   0.397
E N  4IOFA                576    0.505  73.976   0.374   0.502   0.002   0.495
E E  4IOFA                577    0.542  94.705   0.427   0.802   0.014   0.185
B Y  4IOFA                578    0.259  55.284  -0.069   0.879   0.010   0.111
B A  4IOFA                579    0.268  29.523  -0.750   0.879   0.010   0.111
E Y  4IOFA                580    0.328  70.029   0.084   0.879   0.010   0.111
E A  4IOFA                581    0.427  47.066  -0.303   0.879   0.010   0.111
E A  4IOFA                582    0.309  34.008  -0.297   0.831   0.044   0.125
E E  4IOFA                583    0.355  61.949  -0.721   0.701   0.107   0.192
B L  4IOFA                584    0.267  48.833  -0.540   0.453   0.248   0.299
E A  4IOFA                585    0.353  38.857  -0.423   0.252   0.423   0.325
B G  4IOFA                586    0.250  19.675  -0.058   0.085   0.775   0.140
B L  4IOFA                587    0.084  15.307  -1.066   0.018   0.846   0.136
B S  4IOFA                588    0.191  22.409  -1.129   0.011   0.918   0.071
B Y  4IOFA                589    0.107  22.930  -0.430   0.011   0.918   0.071
B D  4IOFA                590    0.123  17.652  -0.995   0.001   0.900   0.099
B L  4IOFA                591    0.075  13.787  -1.328   0.001   0.900   0.099
B Q  4IOFA                592    0.186  33.220  -0.817   0.001   0.900   0.099
B N  4IOFA                593    0.161  23.585  -0.981   0.003   0.718   0.279
B T  4IOFA                594    0.187  25.868  -1.269   0.005   0.336   0.660
E I  4IOFA                595    0.473  87.468  -0.552   0.004   0.197   0.799
E Y  4IOFA                596    0.469 100.161  -1.190   0.004   0.138   0.858
B G  4IOFA                597    0.162  12.734  -0.425   0.003   0.718   0.279
B M  4IOFA                598    0.074  14.827  -0.217   0.001   0.959   0.040
B Y  4IOFA                599    0.070  15.002  -0.011   0.001   0.959   0.040
B L  4IOFA                600    0.029   5.292   0.502   0.000   0.983   0.017
B S  4IOFA                601    0.106  12.376   0.221   0.000   0.983   0.017
B V  4IOFA                602    0.030   4.657  -0.364   0.001   0.959   0.040
B K  4IOFA                603    0.226  46.591   0.292   0.001   0.959   0.040
B G  4IOFA                604    0.032   2.542  -0.681   0.000   0.720   0.280
B Y  4IOFA                605    0.172  36.671   0.746   0.004   0.197   0.799
E N  4IOFA                606    0.332  48.605   0.325   0.257   0.016   0.727
E D  4IOFA                607    0.683  98.377   0.612   0.600   0.003   0.397
E K  4IOFA                608    0.362  74.525   0.710   0.502   0.002   0.495
B Q  4IOFA                609    0.031   5.501   0.887   0.923   0.002   0.076
E P  4IOFA                610    0.350  49.622   1.014   0.970   0.001   0.030
E I  4IOFA                611    0.427  78.958   1.718   0.970   0.001   0.030
B L  4IOFA                612    0.035   6.463  -0.000   0.970   0.001   0.030
B L  4IOFA                613    0.029   5.328   0.954   0.970   0.001   0.030
E K  4IOFA                614    0.426  87.587   1.906   0.970   0.001   0.030
B K  4IOFA                615    0.152  31.328   1.829   0.970   0.001   0.030
B I  4IOFA                616    0.027   5.032   0.608   0.970   0.001   0.030
B I  4IOFA                617    0.112  20.702   0.676   0.988   0.000   0.012
E E  4IOFA                618    0.373  65.111   0.890   0.970   0.001   0.030
B K  4IOFA                619    0.130  26.823   1.285   0.970   0.001   0.030
B M  4IOFA                620    0.043   8.604  -0.479   0.858   0.002   0.139
E A  4IOFA                621    0.363  39.992   0.386   0.600   0.003   0.397
E T  4IOFA                622    0.554  76.881   0.441   0.176   0.004   0.820
B F  4IOFA                623    0.156  31.249   0.498   0.053   0.005   0.942
E E  4IOFA                624    0.636 111.127   0.915   0.003   0.003   0.994
B I  4IOFA                625    0.070  12.932   0.291   0.005   0.015   0.979
E D  4IOFA                626    0.486  70.076   1.895   0.003   0.003   0.994
E E  4IOFA                627    0.617 107.737   1.103   0.923   0.002   0.076
E K  4IOFA                628    0.717 147.405   1.572   0.923   0.002   0.076
E R  4IOFA                629    0.280  64.120   1.657   0.970   0.001   0.030
B F  4IOFA                630    0.056  11.259   0.993   0.970   0.001   0.030
E E  4IOFA                631    0.480  83.786   1.785   0.970   0.001   0.030
E I  4IOFA                632    0.485  89.744   2.017   0.988   0.000   0.012
B I  4IOFA                633    0.107  19.721   1.218   0.970   0.001   0.030
B K  4IOFA                634    0.159  32.768   1.213   0.988   0.000   0.012
E E  4IOFA                635    0.525  91.648   1.406   0.988   0.000   0.012
E A  4IOFA                636    0.391  43.099   1.944   0.988   0.000   0.012
B Y  4IOFA                637    0.056  11.924   1.051   0.988   0.000   0.012
B M  4IOFA                638    0.212  42.361   0.904   0.988   0.000   0.012
E R  4IOFA                639    0.550 125.835   1.230   0.988   0.000   0.012
E S  4IOFA                640    0.305  35.734   0.431   0.988   0.000   0.012
B L  4IOFA                641    0.095  17.449   1.024   0.970   0.001   0.030
E N  4IOFA                642    0.496  72.600   0.947   0.923   0.002   0.076
E N  4IOFA                643    0.520  76.084  -0.166   0.782   0.003   0.216
B F  4IOFA                644    0.266  53.306   0.253   0.600   0.003   0.397
E R  4IOFA                645    0.442 101.172  -0.693   0.406   0.004   0.590
E A  4IOFA                646    0.574  63.255  -1.053   0.246   0.004   0.750
E E  4IOFA                647    0.528  92.294  -1.127   0.109   0.005   0.886
E Q  4IOFA                648    0.487  86.907   0.551   0.053   0.005   0.942
B P  4IOFA                649    0.145  20.590  -2.051   0.858   0.002   0.139
B H  4IOFA                650    0.233  42.401  -0.424   0.923   0.002   0.076
E Q  4IOFA                651    0.349  62.385   0.229   0.975   0.003   0.022
B H  4IOFA                652    0.128  23.210   0.899   0.970   0.001   0.030
B A  4IOFA                653    0.022   2.413   0.167   0.970   0.001   0.030
B M  4IOFA                654    0.151  30.295   0.106   0.970   0.001   0.030
E Y  4IOFA                655    0.316  67.551   0.020   0.970   0.001   0.030
B Y  4IOFA                656    0.114  24.298   0.276   0.970   0.001   0.030
B L  4IOFA                657    0.028   5.200  -0.006   0.970   0.001   0.030
B R  4IOFA                658    0.203  46.487   0.159   0.970   0.001   0.030
E L  4IOFA                659    0.322  58.958  -0.296   0.923   0.002   0.076
B L  4IOFA                660    0.083  15.106  -0.599   0.858   0.002   0.139
B M  4IOFA                661    0.056  11.146  -1.143   0.694   0.003   0.303
E T  4IOFA                662    0.287  39.849  -0.153   0.321   0.003   0.675
E E  4IOFA                663    0.608 106.218  -1.166   0.176   0.004   0.820
E V  4IOFA                664    0.483  74.206  -1.455   0.053   0.005   0.942
E A  4IOFA                665    0.388  42.747  -1.673   0.018   0.019   0.964
B W  4IOFA                666    0.210  50.529  -0.659   0.018   0.019   0.964
E T  4IOFA                667    0.335  46.520  -1.298   0.018   0.019   0.964
E K  4IOFA                668    0.311  63.870  -1.381   0.406   0.004   0.590
E D  4IOFA                669    0.528  76.070  -1.468   0.600   0.003   0.397
E E  4IOFA                670    0.435  75.960  -1.112   0.522   0.016   0.462
B L  4IOFA                671    0.162  29.717   0.632   0.802   0.014   0.185
E K  4IOFA                672    0.284  58.501   0.404   0.923   0.002   0.076
E E  4IOFA                673    0.617 107.842   0.660   0.923   0.002   0.076
B A  4IOFA                674    0.252  27.737   0.775   0.923   0.002   0.076
B L  4IOFA                675    0.032   5.823  -0.074   0.858   0.002   0.139
E D  4IOFA                676    0.591  85.178   1.156   0.694   0.003   0.303
E D  4IOFA                677    0.682  98.233   1.697   0.502   0.002   0.495
B V  4IOFA                678    0.077  11.789   0.890   0.321   0.003   0.675
E T  4IOFA                679    0.432  59.863   1.802   0.115   0.016   0.868
E L  4IOFA                680    0.239  43.688   1.248   0.970   0.001   0.030
E P  4IOFA                681    0.737 104.538   1.329   0.970   0.001   0.030
E R  4IOFA                682    0.308  70.578   1.773   0.970   0.001   0.030
B L  4IOFA                683    0.021   3.827   1.035   0.970   0.001   0.030
E K  4IOFA                684    0.313  64.405   1.882   0.970   0.001   0.030
E A  4IOFA                685    0.558  61.536   1.918   0.970   0.001   0.030
B F  4IOFA                686    0.094  18.866   0.984   0.970   0.001   0.030
B I  4IOFA                687    0.083  15.411   1.232   0.970   0.001   0.030
E P  4IOFA                688    0.555  78.811   1.517   0.970   0.001   0.030
E Q  4IOFA                689    0.509  90.925   1.943   0.923   0.002   0.076
B L  4IOFA                690    0.100  18.292   0.636   0.858   0.002   0.139
B L  4IOFA                691    0.134  24.499   0.546   0.600   0.003   0.397
E S  4IOFA                692    0.501  58.764   0.817   0.181   0.016   0.803
E R  4IOFA                693    0.441 101.058  -0.550   0.058   0.017   0.925
E L  4IOFA                694    0.494  90.525  -0.986   0.018   0.047   0.935
E H  4IOFA                695    0.280  50.950   0.703   0.004   0.197   0.799
B I  4IOFA                696    0.032   5.957  -0.016   0.002   0.816   0.182
B E  4IOFA                697    0.107  18.693   1.151   0.000   0.983   0.017
B A  4IOFA                698    0.017   1.906   1.272   0.000   0.983   0.017
B L  4IOFA                699    0.032   5.786   0.974   0.000   0.983   0.017
B L  4IOFA                700    0.022   3.973   0.721   0.000   0.983   0.017
B H  4IOFA                701    0.059  10.750   0.426   0.001   0.959   0.040
B G  4IOFA                702    0.065   5.100  -0.838   0.004   0.616   0.381
E N  4IOFA                703    0.370  54.168  -0.123   0.004   0.138   0.858
B I  4IOFA                704    0.111  20.498   0.746   0.005   0.015   0.979
E T  4IOFA                705    0.465  64.482   1.427   0.005   0.015   0.979
E K  4IOFA                706    0.325  66.811   1.067   0.923   0.002   0.076
E Q  4IOFA                707    0.752 134.361   0.717   0.970   0.001   0.030
E A  4IOFA                708    0.373  41.127   1.354   0.970   0.001   0.030
B A  4IOFA                709    0.029   3.218  -0.129   0.970   0.001   0.030
E L  4IOFA                710    0.287  52.495   1.659   0.970   0.001   0.030
E G  4IOFA                711    0.600  47.236   1.857   0.970   0.001   0.030
B I  4IOFA                712    0.082  15.078   0.376   0.970   0.001   0.030
B M  4IOFA                713    0.032   6.343   0.119   0.970   0.001   0.030
E Q  4IOFA                714    0.551  98.426   2.030   0.970   0.001   0.030
E M  4IOFA                715    0.467  93.447   1.926   0.970   0.001   0.030
B V  4IOFA                716    0.044   6.809   0.241   0.970   0.001   0.030
B E  4IOFA                717    0.257  44.915   0.165   0.923   0.002   0.076
E D  4IOFA                718    0.729 105.092   1.156   0.782   0.003   0.216
E T  4IOFA                719    0.369  51.125   0.432   0.694   0.003   0.303
B L  4IOFA                720    0.060  11.041   0.053   0.406   0.004   0.590
E I  4IOFA                721    0.525  97.033  -0.152   0.246   0.004   0.750
E E  4IOFA                722    0.707 123.513  -0.309   0.176   0.004   0.820
E H  4IOFA                723    0.392  71.287  -0.621   0.176   0.004   0.820
E A  4IOFA                724    0.605  66.638  -1.994   0.109   0.005   0.886
E H  4IOFA                725    0.649 117.962  -0.841   0.058   0.017   0.925
E T  4IOFA                726    0.325  45.147  -1.435   0.058   0.017   0.925
E K  4IOFA                727    0.452  92.894  -0.661   0.058   0.017   0.925
E P  4IOFA                728    0.573  81.351  -1.261   0.115   0.016   0.868
E L  4IOFA                729    0.276  50.481  -1.145   0.115   0.016   0.868
E L  4IOFA                730    0.371  67.930  -1.293   0.115   0.016   0.868
E P  4IOFA                731    0.499  70.751  -0.971   0.184   0.043   0.773
E S  4IOFA                732    0.525  61.553  -1.173   0.268   0.043   0.689
E Q  4IOFA                733    0.598 106.874  -0.448   0.268   0.043   0.689
E L  4IOFA                734    0.326  59.782  -0.639   0.268   0.043   0.689
B V  4IOFA                735    0.237  36.427  -1.068   0.191   0.086   0.723
E R  4IOFA                736    0.562 128.606  -0.504   0.199   0.152   0.649
E Y  4IOFA                737    0.457  97.746  -1.080   0.199   0.152   0.649
E R  4IOFA                738    0.427  97.760  -0.905   0.125   0.227   0.648
E E  4IOFA                739    0.551  96.330  -1.096   0.066   0.296   0.638
E V  4IOFA                740    0.291  44.788  -0.247   0.069   0.386   0.545
E Q  4IOFA                741    0.618 110.375  -0.873   0.069   0.386   0.545
B L  4IOFA                742    0.192  35.210  -0.733   0.019   0.141   0.840
E P  4IOFA                743    0.482  68.353  -1.238   0.018   0.088   0.893
E D  4IOFA                744    0.587  84.515  -1.371   0.018   0.088   0.893
E R  4IOFA                745    0.547 125.263  -1.180   0.004   0.197   0.799
E G  4IOFA                746    0.424  33.400  -0.568   0.004   0.514   0.481
E W  4IOFA                747    0.390  93.699  -0.090   0.002   0.816   0.182
B F  4IOFA                748    0.157  31.410   0.559   0.001   0.959   0.040
B V  4IOFA                749    0.188  28.849   0.130   0.001   0.959   0.040
B Y  4IOFA                750    0.143  30.538   0.668   0.001   0.959   0.040
E Q  4IOFA                751    0.377  67.243   0.414   0.001   0.959   0.040
B Q  4IOFA                752    0.249  44.525   0.046   0.003   0.718   0.279
E R  4IOFA                753    0.447 102.294  -0.553   0.005   0.336   0.660
E N  4IOFA                754    0.551  80.696  -0.679   0.018   0.088   0.893
E E  4IOFA                755    0.542  94.652  -0.873   0.053   0.043   0.903
E V  4IOFA                756    0.611  93.972  -1.525   0.113   0.043   0.844
E H  4IOFA                757    0.413  75.216  -0.715   0.113   0.087   0.800
E N  4IOFA                758    0.386  56.481  -0.366   0.216   0.235   0.548
B N  4IOFA                759    0.236  34.506  -0.290   0.074   0.484   0.442
B S  4IOFA                760    0.061   7.196   0.320   0.021   0.756   0.223
B G  4IOFA                761    0.037   2.943  -0.193   0.011   0.918   0.071
B I  4IOFA                762    0.025   4.643   0.669   0.011   0.918   0.071
B E  4IOFA                763    0.027   4.752   0.192   0.006   0.962   0.032
B I  4IOFA                764    0.033   6.179  -0.162   0.011   0.918   0.071
B Y  4IOFA                765    0.038   8.163  -0.770   0.018   0.846   0.136
B Y  4IOFA                766    0.099  21.156  -0.049   0.023   0.655   0.322
E Q  4IOFA                767    0.261  46.704  -0.677   0.021   0.279   0.699
B T  4IOFA                768    0.173  24.037  -1.157   0.018   0.047   0.935
E D  4IOFA                769    0.354  50.954  -0.827   0.018   0.019   0.964
E M  4IOFA                770    0.494  98.769  -1.037   0.018   0.019   0.964
E Q  4IOFA                771    0.403  72.065  -1.120   0.018   0.019   0.964
E S  4IOFA                772    0.487  57.053  -0.858   0.016   0.005   0.979
B T  4IOFA                773    0.191  26.547  -0.315   0.600   0.003   0.397
E S  4IOFA                774    0.369  43.200   0.101   0.858   0.002   0.139
B E  4IOFA                775    0.162  28.214  -0.957   0.858   0.002   0.139
B N  4IOFA                776    0.205  29.983   1.005   0.970   0.001   0.030
B M  4IOFA                777    0.057  11.466   0.628   0.970   0.001   0.030
B F  4IOFA                778    0.051  10.196   0.474   0.970   0.001   0.030
B L  4IOFA                779    0.059  10.894  -0.160   0.970   0.001   0.030
B E  4IOFA                780    0.225  39.307   1.241   0.988   0.000   0.012
B L  4IOFA                781    0.064  11.755  -0.298   0.988   0.000   0.012
B F  4IOFA                782    0.060  11.982  -0.387   0.970   0.001   0.030
B A  4IOFA                783    0.201  22.128  -0.952   0.970   0.001   0.030
E Q  4IOFA                784    0.317  56.616  -0.630   0.923   0.002   0.076
B I  4IOFA                785    0.169  31.320  -0.703   0.858   0.002   0.139
B I  4IOFA                786    0.076  13.986  -0.765   0.694   0.003   0.303
B S  4IOFA                787    0.225  26.393  -0.980   0.600   0.003   0.397
B E  4IOFA                788    0.208  36.355  -1.527   0.782   0.003   0.216
E P  4IOFA                789    0.347  49.310   0.949   0.923   0.002   0.076
B A  4IOFA                790    0.041   4.518  -0.322   0.923   0.002   0.076
B F  4IOFA                791    0.110  22.117   0.095   0.938   0.007   0.055
E N  4IOFA                792    0.410  60.039   0.846   0.938   0.007   0.055
E T  4IOFA                793    0.324  44.994   1.612   0.938   0.007   0.055
B L  4IOFA                794    0.026   4.669   1.033   0.938   0.007   0.055
B R  4IOFA                795    0.181  41.403   1.361   0.911   0.033   0.057
E T  4IOFA                796    0.514  71.347   0.537   0.751   0.050   0.199
E K  4IOFA                797    0.531 109.165   0.765   0.561   0.047   0.393
E E  4IOFA                798    0.428  74.684  -0.042   0.184   0.043   0.773
E Q  4IOFA                799    0.307  54.759  -0.928   0.052   0.084   0.864
B L  4IOFA                800    0.153  28.051  -0.099   0.021   0.451   0.528
B G  4IOFA                801    0.025   1.983  -0.841   0.018   0.846   0.136
B Y  4IOFA                802    0.088  18.891  -0.429   0.011   0.918   0.071
B I  4IOFA                803    0.036   6.623  -1.004   0.001   0.900   0.099
B V  4IOFA                804    0.040   6.194  -0.390   0.001   0.900   0.099
B F  4IOFA                805    0.058  11.560  -1.229   0.002   0.816   0.182
B S  4IOFA                806    0.022   2.625  -0.779   0.004   0.616   0.381
B G  4IOFA                807    0.083   6.532  -1.552   0.004   0.514   0.481
B P  4IOFA                808    0.081  11.551  -2.277   0.004   0.420   0.576
B R  4IOFA                809    0.158  36.090  -1.112   0.022   0.359   0.619
B R  4IOFA                810    0.222  50.792  -2.336   0.019   0.141   0.840
E A  4IOFA                811    0.391  43.088  -2.127   0.018   0.088   0.893
E N  4IOFA                812    0.481  70.389  -1.899   0.018   0.019   0.964
E G  4IOFA                813    0.440  34.644  -1.965   0.018   0.047   0.935
B I  4IOFA                814    0.216  39.923  -1.340   0.004   0.197   0.799
B Q  4IOFA                815    0.127  22.700  -1.796   0.005   0.336   0.660
B G  4IOFA                816    0.060   4.698  -1.174   0.004   0.616   0.381
B L  4IOFA                817    0.031   5.768  -1.086   0.001   0.900   0.099
B R  4IOFA                818    0.047  10.717  -0.738   0.001   0.959   0.040
B F  4IOFA                819    0.017   3.372   0.904   0.000   0.983   0.017
B I  4IOFA                820    0.044   8.140   0.219   0.000   0.983   0.017
B I  4IOFA                821    0.028   5.124  -0.450   0.001   0.959   0.040
B Q  4IOFA                822    0.072  12.788  -0.883   0.002   0.816   0.182
B S  4IOFA                823    0.044   5.157  -1.012   0.004   0.420   0.576
E E  4IOFA                824    0.327  57.127  -0.458   0.004   0.138   0.858
E K  4IOFA                825    0.312  64.076  -0.343   0.005   0.015   0.979
E P  4IOFA                826    0.413  58.647   0.364   0.016   0.005   0.979
B P  4IOFA                827    0.107  15.226  -1.081   0.923   0.002   0.076
E H  4IOFA                828    0.665 120.873   0.980   0.923   0.002   0.076
E Y  4IOFA                829    0.354  75.628   1.748   0.923   0.002   0.076
B L  4IOFA                830    0.018   3.369   1.331   0.970   0.001   0.030
E E  4IOFA                831    0.253  44.269   1.155   0.970   0.001   0.030
E S  4IOFA                832    0.573  67.120   1.875   0.970   0.001   0.030
B R  4IOFA                833    0.111  25.511   1.269   0.970   0.001   0.030
B V  4IOFA                834    0.024   3.627   1.201   0.988   0.000   0.012
E E  4IOFA                835    0.413  72.204   2.121   0.970   0.001   0.030
E A  4IOFA                836    0.395  43.584   2.140   0.988   0.000   0.012
B F  4IOFA                837    0.030   6.121   0.568   0.970   0.001   0.030
B L  4IOFA                838    0.077  14.135   1.344   0.970   0.001   0.030
E I  4IOFA                839    0.516  95.442   1.974   0.970   0.001   0.030
E T  4IOFA                840    0.314  43.538   2.100   0.970   0.001   0.030
B M  4IOFA                841    0.025   4.902   0.946   0.970   0.001   0.030
E E  4IOFA                842    0.323  56.411   1.475   0.970   0.001   0.030
E K  4IOFA                843    0.631 129.797   1.663   0.923   0.002   0.076
B S  4IOFA                844    0.199  23.311   0.906   0.858   0.002   0.139
B I  4IOFA                845    0.145  26.843  -0.124   0.600   0.003   0.397
E E  4IOFA                846    0.715 124.841   1.238   0.321   0.003   0.675
E D  4IOFA                847    0.692  99.688   1.390   0.109   0.005   0.886
B M  4IOFA                848    0.053  10.525   0.936   0.016   0.005   0.979
E T  4IOFA                849    0.378  52.387   1.808   0.016   0.005   0.979
E E  4IOFA                850    0.609 106.410   1.583   0.923   0.002   0.076
E E  4IOFA                851    0.660 115.267   1.956   0.975   0.003   0.022
B A  4IOFA                852    0.166  18.293   1.264   0.975   0.003   0.022
B F  4IOFA                853    0.044   8.891   0.782   0.970   0.001   0.030
E Q  4IOFA                854    0.476  85.014   2.137   0.970   0.001   0.030
E K  4IOFA                855    0.462  95.075   2.376   0.970   0.001   0.030
B H  4IOFA                856    0.049   8.859   0.745   0.970   0.001   0.030
B I  4IOFA                857    0.130  24.050   0.856   0.970   0.001   0.030
E Q  4IOFA                858    0.511  91.193   1.522   0.970   0.001   0.030
E A  4IOFA                859    0.360  39.639   1.427   0.970   0.001   0.030
B L  4IOFA                860    0.067  12.323   0.548   0.970   0.001   0.030
B A  4IOFA                861    0.201  22.194   0.477   0.970   0.001   0.030
E I  4IOFA                862    0.504  93.148   0.083   0.923   0.002   0.076
E R  4IOFA                863    0.373  85.371   0.206   0.923   0.002   0.076
B R  4IOFA                864    0.178  40.831   0.468   0.782   0.003   0.216
E L  4IOFA                865    0.351  64.231  -0.222   0.694   0.003   0.303
E D  4IOFA                866    0.475  68.462  -0.720   0.502   0.002   0.495
E K  4IOFA                867    0.412  84.790  -0.285   0.176   0.004   0.820
E P  4IOFA                868    0.372  52.744  -1.212   0.246   0.004   0.750
E K  4IOFA                869    0.608 125.148  -1.204   0.246   0.004   0.750
E K  4IOFA                870    0.376  77.302  -0.243   0.058   0.017   0.925
B L  4IOFA                871    0.200  36.602  -1.202   0.923   0.002   0.076
E S  4IOFA                872    0.387  45.345  -0.950   0.970   0.001   0.030
E A  4IOFA                873    0.417  45.931  -0.037   0.970   0.001   0.030
B E  4IOFA                874    0.117  20.422   0.367   0.970   0.001   0.030
B S  4IOFA                875    0.023   2.731  -0.717   0.970   0.001   0.030
B A  4IOFA                876    0.217  23.880  -0.088   0.970   0.001   0.030
E K  4IOFA                877    0.267  54.860   0.305   0.970   0.001   0.030
B Y  4IOFA                878    0.043   9.210  -0.099   0.970   0.001   0.030
B W  4IOFA                879    0.057  13.636  -0.294   0.970   0.001   0.030
B G  4IOFA                880    0.210  16.551  -0.063   0.970   0.001   0.030
B E  4IOFA                881    0.100  17.453  -0.477   0.970   0.001   0.030
B I  4IOFA                882    0.048   8.806  -0.619   0.970   0.001   0.030
B I  4IOFA                883    0.185  34.225  -0.509   0.923   0.002   0.076
E S  4IOFA                884    0.384  44.958  -0.476   0.694   0.003   0.303
E Q  4IOFA                885    0.446  79.709  -0.863   0.176   0.004   0.820
E Q  4IOFA                886    0.550  98.194  -1.363   0.016   0.005   0.979
B Y  4IOFA                887    0.154  32.867  -0.004   0.018   0.019   0.964
E N  4IOFA                888    0.446  65.338  -0.094   0.058   0.017   0.925
B F  4IOFA                889    0.209  41.846  -0.281   0.321   0.003   0.675
E D  4IOFA                890    0.605  87.224  -1.203   0.406   0.004   0.590
E R  4IOFA                891    0.282  64.532   0.939   0.600   0.003   0.397
B D  4IOFA                892    0.141  20.347   0.521   0.858   0.002   0.139
E N  4IOFA                893    0.508  74.342   1.023   0.923   0.002   0.076
E T  4IOFA                894    0.530  73.580   1.787   0.970   0.001   0.030
B E  4IOFA                895    0.088  15.391   1.295   0.970   0.001   0.030
B V  4IOFA                896    0.134  20.642   1.042   0.970   0.001   0.030
E A  4IOFA                897    0.625  68.853   1.546   0.970   0.001   0.030
E Y  4IOFA                898    0.269  57.571   1.896   0.970   0.001   0.030
B L  4IOFA                899    0.028   5.090   0.299   0.923   0.002   0.076
E K  4IOFA                900    0.511 105.195   1.651   0.782   0.003   0.216
E T  4IOFA                901    0.669  92.776   1.996   0.406   0.004   0.590
B L  4IOFA                902    0.060  10.931   1.241   0.176   0.004   0.820
E T  4IOFA                903    0.380  52.664   1.994   0.016   0.005   0.979
E K  4IOFA                904    0.294  60.517   1.776   0.970   0.001   0.030
E E  4IOFA                905    0.707 123.426   1.562   0.975   0.003   0.022
B D  4IOFA                906    0.251  36.140   1.936   0.975   0.003   0.022
B I  4IOFA                907    0.021   3.811   1.404   0.970   0.001   0.030
E I  4IOFA                908    0.267  49.432   2.222   0.970   0.001   0.030
E K  4IOFA                909    0.534 109.864   2.124   0.970   0.001   0.030
B F  4IOFA                910    0.076  15.313   1.142   0.988   0.000   0.012
B Y  4IOFA                911    0.023   4.851   1.036   0.988   0.000   0.012
E K  4IOFA                912    0.431  88.718   2.118   0.970   0.001   0.030
E E  4IOFA                913    0.524  91.595   2.162   0.970   0.001   0.030
B M  4IOFA                914    0.110  21.991   1.195   0.923   0.002   0.076
B L  4IOFA                915    0.051   9.411   1.044   0.600   0.003   0.397
E A  4IOFA                916    0.423  46.593   1.896   0.246   0.004   0.750
E V  4IOFA                917    0.590  90.668   0.107   0.176   0.004   0.820
E D  4IOFA                918    0.781 112.600  -0.747   0.016   0.005   0.979
E A  4IOFA                919    0.459  50.637  -0.817   0.018   0.019   0.964
E P  4IOFA                920    0.396  56.164  -0.885   0.018   0.047   0.935
E R  4IOFA                921    0.350  80.196   0.020   0.005   0.262   0.733
B R  4IOFA                922    0.179  41.014   0.208   0.003   0.718   0.279
B H  4IOFA                923    0.099  18.099  -0.131   0.001   0.900   0.099
B K  4IOFA                924    0.114  23.532   0.639   0.001   0.959   0.040
B V  4IOFA                925    0.019   2.859   1.214   0.001   0.959   0.040
B S  4IOFA                926    0.023   2.672   0.844   0.000   0.983   0.017
B V  4IOFA                927    0.020   3.013   0.947   0.000   0.983   0.017
B H  4IOFA                928    0.041   7.385   1.010   0.000   0.983   0.017
B V  4IOFA                929    0.022   3.320   0.670   0.001   0.959   0.040
B L  4IOFA                930    0.123  22.576   0.702   0.003   0.718   0.279
B A  4IOFA                931    0.056   6.160  -0.647   0.004   0.197   0.799
E R  4IOFA                932    0.528 120.866   0.587   0.018   0.019   0.964
E E  4IOFA                933    0.555  96.889  -0.277   0.115   0.016   0.868
E M  4IOFA                934    0.551 110.355  -0.546   0.181   0.016   0.803
E D  4IOFA                935    0.565  81.388   0.177   0.339   0.016   0.645
E S  4IOFA                936    0.381  44.630  -0.641   0.522   0.016   0.462
E N  4IOFA                937    0.480  70.228  -0.222   0.694   0.003   0.303
E P  4IOFA                938    0.548  77.719   0.286   0.717   0.014   0.269
E V  4IOFA                939    0.404  62.110   0.443   0.802   0.014   0.185
B V  4IOFA                940    0.160  24.531  -0.475   0.717   0.014   0.269
E G  4IOFA                941    0.616  48.487  -0.077   0.717   0.014   0.269
E E  4IOFA                942    0.511  89.342   0.335   0.622   0.015   0.363
B F  4IOFA                943    0.206  41.404  -0.436   0.430   0.016   0.555
E P  4IOFA                944    0.472  67.034  -1.357   0.257   0.016   0.727
E A  4IOFA                945    0.410  45.149  -0.638   0.268   0.043   0.689
E Q  4IOFA                946    0.476  85.067  -1.104   0.191   0.086   0.723
E N  4IOFA                947    0.342  50.113  -0.598   0.216   0.235   0.548
E D  4IOFA                948    0.572  82.483  -0.449   0.125   0.227   0.648
B I  4IOFA                949    0.246  45.455  -0.555   0.125   0.227   0.648
E N  4IOFA                950    0.385  56.423  -0.118   0.135   0.317   0.548
B L  4IOFA                951    0.212  38.890   0.301   0.135   0.317   0.548
E S  4IOFA                952    0.595  69.687   0.088   0.135   0.317   0.548
E Q  4IOFA                953    0.488  87.121   0.333   0.135   0.317   0.548
B A  4IOFA                954    0.105  11.604  -0.228   0.118   0.150   0.732
E P  4IOFA                955    0.561  79.592  -0.425   0.113   0.087   0.800
E A  4IOFA                956    0.544  59.982   0.079   0.113   0.043   0.844
B L  4IOFA                957    0.240  43.962  -0.260   0.053   0.043   0.903
E P  4IOFA                958    0.590  83.664  -0.253   0.018   0.019   0.964
E Q  4IOFA                959    0.613 109.410   0.149   0.053   0.043   0.903
E P  4IOFA                960    0.421  59.768  -0.440   0.064   0.216   0.721
E E  4IOFA                961    0.500  87.333   0.250   0.023   0.655   0.322
E V  4IOFA                962    0.420  64.493   1.639   0.021   0.756   0.223
B I  4IOFA                963    0.045   8.325  -0.163   0.023   0.655   0.322
E Q  4IOFA                964    0.515  92.033   0.609   0.069   0.386   0.545
E N  4IOFA                965    0.343  50.230   0.391   0.191   0.086   0.723
B M  4IOFA                966    0.093  18.509   0.878   0.858   0.002   0.139
E T  4IOFA                967    0.550  76.257   0.285   0.879   0.010   0.111
E E  4IOFA                968    0.358  62.490   1.718   0.879   0.010   0.111
B F  4IOFA                969    0.059  11.741   0.711   0.831   0.044   0.125
E K  4IOFA                970    0.338  69.465   0.717   0.751   0.050   0.199
E R  4IOFA                971    0.611 140.034   0.509   0.561   0.047   0.393
E G  4IOFA                972    0.581  45.725  -0.095   0.268   0.043   0.689
B L  4IOFA                973    0.177  32.445   0.916   0.052   0.084   0.864
E P  4IOFA                974    0.476  67.488  -0.584   0.056   0.142   0.802
B L  4IOFA                975    0.120  21.935  -0.952   0.056   0.142   0.802
B F  4IOFA                976    0.200  40.120   0.131   0.118   0.150   0.732
E P  4IOFA                977    0.362  51.325  -1.157   0.113   0.087   0.800
B L  4IOFA                978    0.210  38.378  -0.659   0.113   0.043   0.844
B V  4IOFA                979    0.262  40.254  -0.777   0.184   0.043   0.773
E K  4IOFA                980    0.422  86.826   0.293   0.184   0.043   0.773
B P  4IOFA                981    0.210  29.742  -1.121   0.257   0.016   0.727
B H  4IOFA                982    0.194  35.361   0.199   0.430   0.016   0.555
E I  4IOFA                983    0.410  75.776   0.248   0.600   0.003   0.397
E N  4IOFA                984    0.454  66.451  -0.184   0.717   0.014   0.269
B F  4IOFA                985    0.104  20.893   0.578   0.717   0.014   0.269
E M  4IOFA                986    0.320  64.012   0.710   0.717   0.014   0.269
E A  4IOFA                987    0.315  34.735  -0.656   0.751   0.050   0.199
E A  4IOFA                988    0.494  54.395  -0.304   0.561   0.047   0.393
E K  4IOFA                989    0.606 124.736   0.752   0.354   0.048   0.598
E L  4IOFA                990    0.617 112.899  -0.809   0.003   0.003   0.994

Explain the output.  Go back.
```

---
